# Everybody's Page

**Published:** 1891-04-25

**Authors:** 


### EVERYBODY'S PAGE.

The discoveries of Pasteur and Koch, says the New York *Medical Record*, render necessary the coining of some new words; one of these is the adjective "immune." The *Record* suggests that the corresponding verb shall be "to immunate," which is neat, and goes well with "to vaccinate."

Dr. Danford Thomas at the monthly meeting of the Public Sanitary Inspectors' Association lectured on burial and funeral reform. He advocated earth to earth system, the early interment of bodies, the interment in suitable ground and perishable coffins, and said that only one adult body should be interred in one grave until at any rate sufficient time was given for its complete amalgamation with the earth. But these precautions do not reduce the ever-increasing difficulty of want of space, with the accompanying increase of population; for these there can be no method but cremation.

It has been said lately in New York that as many of the ready-made clothes are made in overcrowded dirty tenement houses it is very probable that these same clothes distribute disease germs of most kinds about the country. This is more than probable; it is very likely. A reach-me-down garment has its advantages, but it may prove a doubtful blessing if it hails from a scarlet-fever vicinity.

Even cremation will have its drawbacks if many people leave commands in their will similar to those lately left by a physician in Baltimore. His will directed that, owing to his horror of being buried alive, his heart after death should be removed before witnesses, and that his body should be cremated and the ashes distributed to his friends in silver phials. There is something very uncomfortable and disrespectful even in the idea of possessing a little bit of an old friend; personally, we think portraits preferable mementos.

